# Owner Social Determinants of Health Associated with Exercise Patterns in Golden Retrievers with and Without Cancer

**DOI:** 10.3390/vetsci13020172

**Published:** 2026-02-09

**Authors:** Elpida Artemiou, Andrea Paredes, Sarah Hooper

**Affiliations:** 1 School of Veterinary Medicine, Texas Tech University, Amarillo, TX 79106, USA; andrpare@ttu.edu; 2 College of Veterinary Medicine, Arkansas State University, Jonesboro, AR 72467, USA

**Keywords:** canine exercise, canine cancer, canine physical activity, companion animal medicine, golden retriever, golden retriever lifetime study, GPBoost Poisson models, Morris Animal Foundation, social determinants of health (SDHs), social determinants of animal health (SDAHs)

## Abstract

The World Health Organization (WHO) recognizes the impact of social determinants of health (SDHs) on human health and wellbeing factors. Limited research has explored how SDHs, such as the social, economic, and environmental conditions in which individuals are born, live, work, and grow older, shape exercise behaviors and chronic health conditions such as cancer in dogs. This study links SDHs identified through owner-provided continental United States zip codes with levels of physical activity. We hypothesized that owners with higher incomes, education, and access to healthcare services positively influence their dog’s health outcomes, specifically owner-reported physical activity. Our study utilized all owner-provided data, collected between 2012 and 2022, from the first seven years of owner surveys for the 3044 Golden Retrievers enrolled in the Morris Animal Foundation Lifetime Study. Sixteen GPBoost Poisson models were built to assess the impact of twenty-three social determinants in Golden Retrievers with and without a diagnosis of cancer. SHAP values were calculated for each dependent variable. Consistently, economic factors, education, ethnicity, and health care access were identified as important variables. Furthermore, our findings suggest that complex interactions between ethnicities and other SDHs should be explored in future studies.

## Simple Summary

This study explores how social determinants of health (SDHs), the non-medical factors that influence a person’s health and wellbeing, also impact exercise behaviors and chronic health conditions such as cancer in dogs. We used owner-provided zip codes along with survey data to evaluate if owners with higher incomes, education levels, and increased access to healthcare services positively influenced their dog’s health outcomes. We used all 3044 Golden Retriever owner-provided data from the first seven years of Golden Retriever Lifetime Study owner surveys. We built sixteen prediction models to assess the impact of twenty-three social determinants in Golden Retrievers with and without cancer. We assessed which factors had the biggest influence on the results, using a method that shows how much each factor matters. Consistently, economic factors, education, ethnicity, and health care access were identified as important variables. Furthermore, our results also highlight the need for future research to better understand how ethnicity interacts with other SDHs.

## 1. Introduction

Social Determinants of Health (SDHs) refer to a broad range of factors, including “social, economic, and environmental conditions in which individuals are born, live, work, and grow older” [1]. These determinants impact quality of life, overall health, and risks of disease and potentially contribute to early death [1]. SDHs identify factors such as employment conditions and income; access to and level of education, including language and literacy skills; housing; neighborhood environments, including whether individuals live in rural or urban communities; reliable transportation; healthcare services; the availability of social support networks; food security; gender; and more [2]. Evidence from human medicine shows a strong relationship between food and housing insecurity and reduced medication adherence, which can lead to negative health outcomes, particularly in individuals managing long term chronic conditions [3]. Education, including both access to and level of education, is another critical SDH that is directly linked to income levels and access to stable health insurance, making it a foundational factor in promoting overall health outcomes and cancer survival [4,5]. Not surprisingly, it has been shown that individuals that live in low-income communities experience significant health disadvantages, which can also be associated with shorter life expectancy [1,6]. The development of cancer is one contributor to this shorter life expectancy. There are strong relationships between income levels, disadvantaged neighborhoods, and home types, as well as other SDHs such as access to reliable transportation and renting a home, which have been documented to impact human cancer risk, treatment, and survival [5,7,8].

Because companion animals share their environments and lifestyles with their owners, SDHs may similarly influence canine cancer risk. The 2024 AVMA Pet Ownership and Demographic Sourcebook reports that there are 89.7 million dogs and 73.8 million cats in the U.S., with 45.5% of households owning a dog and 32.1% owning a cat [9]. Additional evidence shows that 97% of pet owners consider their pets to be part of the family, with nearly half viewing them as equivalent to a human family member [10]. This interconnection is further reflected in the way that SDHs that affect human health and well-being often parallel the challenges experienced in animals, impacting both their health and welfare [1,6]. Evidence from rural, remote, and low-income communities highlights the pressing need for additional and affordable veterinary services. These areas frequently report a higher number of stray animals and animal shelter intake, increased transmission of zoonotic diseases, elevated rates of preventable diseases, and premature animal deaths [11]. Blackwell et al., 2023 [12] reported that one out of four households in the United States had not sought veterinary care for a period of two years due to issues with affordability. A study using data from the Dog Aging Project (DAP) investigated age-related changes in canine health, mobility, and disease in relation to the animal’s social and environmental conditions. The findings of the study revealed that higher household income, social companionship, and environmental enrichment were associated with improved health outcomes in dogs. Interestingly, a positive correlation was observed between an owner’s wealth and the number of diseases diagnosed. The study also found that younger dogs living with older owners had better health outcomes compared to older dogs with older owners. Conversely, younger dogs that lived with owners that experienced housing insecurity exhibited poorer health outcomes compared to older dogs living in secure and stable environments. As expected, older dogs in high-income households were reported to be in better health than those in lower income households; however, income was a less significant predictor of health outcomes in younger animals. Additionally, the neighborhood environment influenced mobility, with older dogs experiencing reduced mobility when living in less stable communities [13].

Given these links between social determinants and health outcomes in both humans and animals, examining modifiable behaviors is essential and may give insight on how to develop effective strategies to improve health and reduce cancer risks by changing behaviors. It is well established that owning a dog can influence an owner’s behavior by increasing physical activity, specifically the amount of time spent walking [14]. The promotion of dog walking has been used as a strategy in several interventional studies with outcomes suggesting these strategies are effective in some populations for increasing and maintaining regular physical activity [15]. Increased physical activity has been shown to reduce the risk of certain types of cancer in humans by up to 50 percent [16], in part due to a reduction in obesity, one of the major risk factors for developing cancer [17].

Beyond cancer, it is widely recognized that physical activity has protective effects against chronic illness [18,19], with financial factors, owner age, household stability, and social time being SDHs identified as increasing physical activity in humans [20,21,22,23] as well as in dogs enrolled in the Dog Aging Project [10]. However, physical activity performed outdoors may increase exposure to environmental risk factors such as ultraviolet radiation (UV). A human case-controlled study showed that increased physical activity was linked to an increased melanoma risk, which the authors hypothesized to be from greater incidental sun exposure given that the physical activity–melanoma association was stronger in high-UV areas [24].

However, limited research has explored how SDH shapes exercise behaviors in dogs and their owners—such as frequency and type of physical activity—and whether these patterns are associated with chronic health conditions such as cancer. This study utilized SDHs based on owner-provided zip codes linking to physical activity in cancer-free and cancer-diagnosed Golden Retrievers enrolled in the Morris Animal Foundation (MAF) Golden Retriever Lifetime Study (GRLS). We hypothesized that owners with higher income, greater education level, and better access to healthcare services would self-report higher levels of canine physical activity, which would be linked with improved animal health outcomes, including a greater likelihood of remaining cancer-free, and an increased lifespan among Golden Retrievers diagnosed with cancer enrolled in the study.

## 2. Materials and Methods

### 2.1. Ethics

The MAF GRLS received approval from the Morris Animal Foundation Animal Welfare Advisory Board (AWAB) under grant ID D10CLP-001 and approval code MAF-100. Full AWAB reviews are conducted every three years. The most recent approval was granted on 11 December 2024, with the original approval date of 9 June 2011 [25]. Informed consent was obtained from all participating owners and veterinarians and, prior to enrollment, both owners and their veterinarians were required to agree to complete each year an online survey, document the veterinary annual physical exam findings, and submit biological samples to MAF.

### 2.2. Study Design

The MAF GRLS is a prospective, observational, longitudinal cohort study. As previously described by Guy et al. (2015) [25], healthy, purebred Golden Retrievers were eligible to enroll if they met the following criteria: (1) were privately owned, (2) were between six months and two years of age at enrollment, (3) were located within the contiguous United States, and (4) the pedigrees were documented for a minimum of two generations.

Enrolled Golden Retrievers are being followed for their lifetime in an effort to identify the incidence and important risk factors of common canine cancers as well as other canine diseases [25]. The first Golden Retriever was enrolled in 2012, and the last Golden Retriever was enrolled in 2015. The study remains ongoing in 2025.

### 2.3. Study Population

Our study incorporated records of all 3044 purebred Golden Retrievers enrolled in the MAF GRLS during years zero through seven. The MAF GRLS data included in this study was collected through owner surveys during 2012 through 2022.

### 2.4. Raw Activity Data and BiMM Forest Development

All raw data, data preparation, preprocessing, and development of the Binary Mixed Models (BiMM) with random forest models is fully described by Dennis and Hooper et al. (2025) [26]. In brief, the “Dog Demographics”, “Activity and Lifestyle”, and “Conditions Neoplasia” datasets were obtained from MAF’s Data Commons Portal. The “Activity and Lifestyle” dataset is collected from the Annual Owner Questionnaire [27]. The “Conditions Neoplasia” dataset is collected from information reported in the Annual Veterinarian Questionnaire, Additional Veterinarian Visit Questionnaire, Malignancy Related Questionnaire, and Death and Necropsy Related Questionnaire [28]. Owner-reported questions with <50% response rates were excluded. Due to question changes in the MAF survey in year three, we standardized variable formats across years and retained only data from questions held consistent over years zero through seven or added in year three. After merging the datasets, duplicates were removed and free-text “other” activity entries were manually reviewed and recoded into existing categories or grouped under a new “other activities” variable. All variables were converted to numeric variables using ordinal or one-hot encoding. Missing data was imputed using multivariate imputation by chained equations (MICE) with random forests, performing five imputations with 30 iterations each. To balance class distributions, Synthetic Minority Over-Sampling Technique for Nominal and Continuous (SMOTE-NC) with a k value of 7 was applied. All continuous variables were scaled. BiMM random forest models were built using H3 methodology and the R code from Speiser et al. (2019), with an error tolerance of 0.01 and one iteration [29]. Models were run separately on each of the five imputations for both datasets, and the results were pooled into a single confusion matrix. Final performance metrics, including accuracy, sensitivity, specificity, precision, F1 score, and AUC with 95% confidence intervals, were calculated from this matrix.

### 2.5. Social Determinants of Health Data Preparation and Preprocessing

Under a data agreement with MAF, we obtained information about each dog’s role (e.g., breeder or companion pet) and lifestyle, including owner’s home type and age, heating and cooling systems, water source, and the state and zip code of the owner’s primary residence (Table 1). We used the year of study as a proxy for lifespan, since the study remains ongoing at the time of this study. We grouped the 24 reported dog lifestyle categories into eight broader groups: agility/sport dogs, breeder, companion pet, hunting, multiple uses, show, therapy dog, or working dog.

We obtained the median and mean income (Table S1901), percentage of owners that are bachelor’s degree holders and high school graduates (Table S1501), percentage of owners without healthcare (Table S2701), and race demographics (Table DP05) from the 2023 US Census Bureau American Community Survey (ACS) [30], 5-year estimates, for each owner’s zip code.

Since weather has been documented to impact the amount of physical activity in humans [31], states were further classified based on three temperature categories using the National Oceanic and Atmospheric Administration (NOAA) Northeast Regional Climate Center’s Gridded Normals Mapper [32], with the region was set to CONUS, the element set to average temperature and date set to season/year annual. States were classified as hot temperature states (average temperatures greater than 60 °F), mild temperature states (average temperatures 50 °F to 60 °F), or cold temperature states (average temperatures less than 50 °F). After preparation and preprocessing, we merged all social determinant data with the activity dataset previously described [26].

The lifestyle category, primary state of residence, geographical delineation (e.g., rural environment), water supply, home type, and temperature of the state were one hot encoded using the caret R-package version 7.0.1 [33]. Duplicate columns were removed.

### 2.6. Social Determinants of Health Data Modeling

We divided the dataset into two groups based upon if cancer occurred in the lifetime: Golden Retrievers that were never diagnosed with a cancer diagnosis and Golden Retrievers that were diagnosed with cancer, with all available data placed into this dataset regardless of when the cancer diagnosis occurred. For each group, we constructed Poisson GPBoost models using the R-package GPBoost version 1.5.6 [34]. The dependent variables in the analysis were the social determinants of health, while the independent variables included year in study; frequency, pace, and duration of exercise; frequency of cold- and warm-water swimming; activity level; and duration of sun exposure. These eight variables were previously identified by Dennis and Hooper (2025) as the most important physical activity-related predictors of neoplasia development in Golden Retrievers [26]. We selected predictors based on a mean decrease in Gini value greater than 2000.

We built 16 GPBoost models—eight for Golden Retrievers without a cancer diagnosis and eight for Golden Retrievers with a cancer diagnosis—each assessing one of the independent variables. For each Poisson GPBoost model, the dataset was divided into a training set, which contained 50–70% of each level of the physical activity variable (depending on the variable response distribution), and a testing set comprising the remaining 30–50% of the data. Golden Retriever subjects contained within the testing set were not included in the training set. We used the Golden Retrievers unique subject id for the group data and cluster_ids parameters within each model. Using the predicted and actual data, the mean squared error (MSE) and the root mean squared error (RMSE) were calculated for each model. We constructed null models to calculate McFaddon’s R^2^.

After construction of each model, we performed a hyperparameter grid search using the tuning function within the GPBoost R-package. Based upon the initial model performance, between 10 and 1000 trees were constructed for each of the 1000 to 1,000,000 random grid search parameters calculated. We calculated the MSE, RMSE, and McFaddon’s R^2^ for the tuned models and retained the parameters from the best performing model (Appendix A). For each best performing model, we used the R-package SHAPforxgboost version 0.1.3 [35] to calculate the SHAP values and produce summary plots for each model. The R code for the analysis is available in the Github Repository MicroBatVet/GRLS [36].

## 3. Results

We excluded 267 records—approximately 1% of the total SDH data obtained from MAF—due to incomplete owner zip codes. The full BiMMs random forest results were previously reported by Dennis and Hooper et al. (2025) [26]. The full demographic data was also previously reported by Dennis and Hooper et al. (2025) [26]. In brief, 219 intact females, 431 intact males, 1109 neutered males, and 1285 spayed females were enrolled at baseline (year 0). Two-hundred and seventy-seven enrolled dogs were diagnosed with cancer, with the medial age of a cancer diagnosis occurring at 6.1 years.

### 3.1. SDH for Year in Study

**Figure 1:**
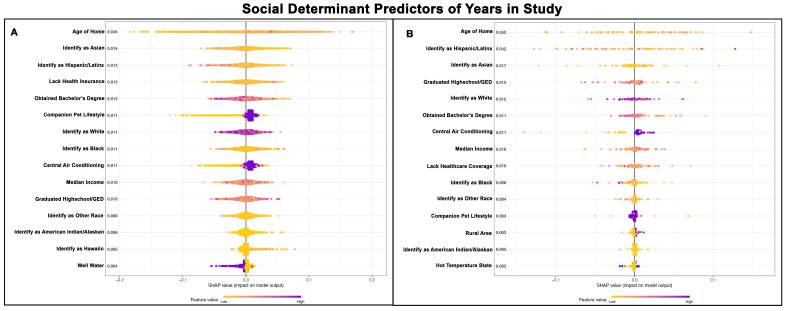
SHAP summary plots comparing SDH feature importance for years in study in Golden Retrievers without a cancer diagnosis (**A**) and with a cancer diagnosis (**B**). The features are ranked top-to-bottom by mean absolute SHAP value, with more important predictors at the top and descending in order of importance. Each point represents an individual Golden Retriever, with low values represented in yellow and high values in purple.

The Poisson GPBoost models predicting year of study performed well, with McFadden’s Pseudo R^2^ values of 0.70 for Golden Retrievers without a cancer diagnosis (Table 2) and 0.80 for Golden Retrievers with a cancer diagnosis (Table 3). In both groups, the age of the home was the top predictor (Figure 1A,B). For Golden Retrievers without cancer, areas with higher percentages of Asian and Hispanic/Latinx populations tended to participate more in the earlier study years and less in the later study years (Figure 1A), indicating those Goldens from areas with lower Asian and Hispanic/Latinx populations were more likely to remain in the study. In the cancer diagnosed Golden Retriever group, the impact of the percentage of the population identifying as Hispanic/Latinx was greater, ranking as the second most important predictor (Figure 1B). The earlier years of the study were also closely linked to populations with a higher percentage of the population which earned bachelor’s degrees for both populations of Golden Retrievers (Figure 1A,B). The companion pet lifestyle was identified as the 6th most important predictor for Golden Retrievers without cancer and suggests those Goldens who served as companion pets were more likely to remain in the study (Figure 1A). This pattern does not appear in Golden Retrievers diagnosed with cancer (Figure 1B). Golden retrievers living in households with central air conditioning were more likely to stay enrolled in the study (Figure 1A,B). Other important variables such as race, demographics, and median income showed less interpretable patterns across the two groups (Figure 1A,B).

### 3.2. SDH for Frequency of Exercise

**Figure 2:**
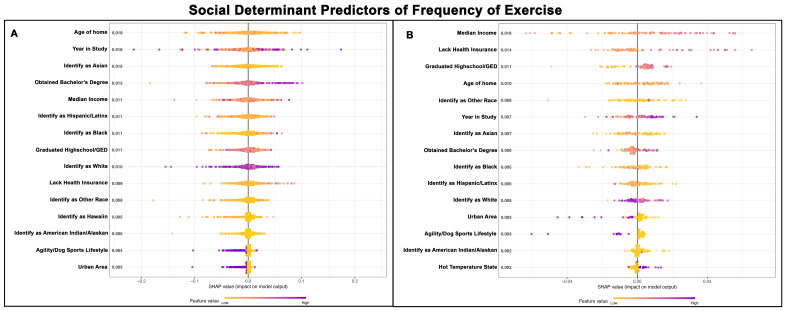
SHAP summary plots comparing SDH feature importance for frequency of exercise in Golden Retrievers without a cancer diagnosis (**A**) and with a cancer diagnosis (**B**). The features are ranked top-to-bottom by mean absolute SHAP value, with more important predictors at the top and descending in order of importance. Each point represents an individual Golden Retriever, with low values represented in yellow and high values in purple.

The Poisson GPBoost models had excellent performance, explaining approximately 94% of the variance in exercise frequency based on the SDHs (Table 2 and Table 3). For Golden Retrievers without cancer, the age of the home emerged as the most important predictor, followed by the year of study (Figure 2A). The percentage of the population identifying as Asian was identified as the third most important predictor for Golden Retrievers without a cancer diagnosis. Golden Retrievers were exercised less frequently if residing in an area with a higher percentage of Asian-identifying individuals (Figure 2A). In contrast, dogs living in areas reporting a higher percentage of adults with bachelor’s degrees were exercised more frequently (Figure 2A). Median income was the fifth most important predictor of Golden Retrievers without cancer but was the most important predictor of frequency of exercise in Golden Retrievers with a cancer diagnosis (Figure 2A,B). Golden retrievers with a cancer diagnosis were exercised more frequently in areas with higher median salaries (Figure 2B). The percentage of individuals who graduated high school or earned a GED (General Educational Development) was the eighth most important predictor of exercise frequency for Golden Retrievers without a cancer diagnosis (Figure 2A), but it was the third most important predictor for Goldens with a cancer diagnosis (Figure 2B). Golden Retrievers with a cancer diagnosis and residing in areas with higher high school graduation rates were exercised more frequently (Figure 2B). Certain predictors, such as urban areas, were clearly associated with a lower frequency of exercise (Figure 2A,B).

Golden Retrievers with cancer were walked more frequently living in areas where a higher percentage of the population lacked health insurance and areas with a higher percentage of the population having graduated high school or earned a GED (Figure 2B). While the age of the home was the fourth most important predictor in the cancer group, no clear trend emerged regarding its relationship with exercise frequency based upon the Shapley values. Golden Retrievers with cancer were more likely to come from areas with a low percentage of individuals identifying as a race not listed in the ACS. Golden Retrievers with cancer also appeared to be walked less frequently if they lived in areas with higher Asian, Black, and White populations, whereas dogs were walked more frequently in areas with higher American Indian and Native Alaskan populations (Figure 2B). Later years of the study tended to be associated with increased exercise frequency. Figure 2A,B show the top 15 SDH predictors of exercise frequency for Golden Retrievers without cancer and those with cancer, respectively.

### 3.3. SDH for Pace of Exercise

The Poisson GPBoost model predicting the pace of exercise for Golden Retrievers without a cancer diagnosis exhibited a good fit, with a McFadden’s Pseudo R^2^ value of 0.24 (Table 2). In contrast, the model predicting the pace of exercise for Golden Retrievers with a cancer diagnosis did not perform well, with a McFadden’s Pseudo R^2^ value of 0.07 (Table 3).

**Figure 3:**
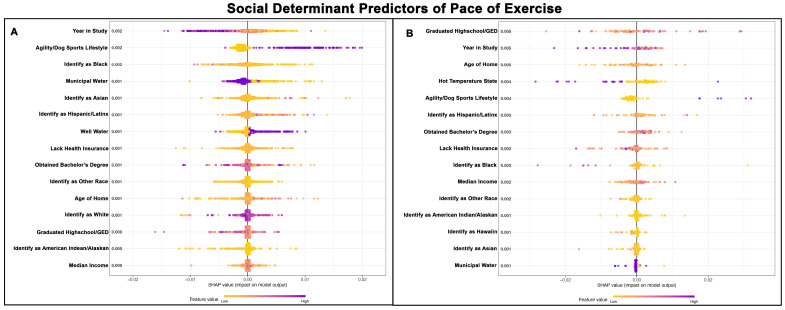
SHAP summary plots comparing SDH feature importance for pace of exercise in Golden Retrievers without a cancer diagnosis (**A**) and with a cancer diagnosis (**B**). The features are ranked top-to-bottom by mean absolute SHAP value, with more important predictors at the top and descending in order of importance. Each point represents an individual Golden Retriever, with low values represented in yellow and high values in purple.

Thirteen of the top fifteen predictors for pace of exercise were the same for both groups of Golden Retrievers, though the order of their relative importance varied (Figure 3A,B). For Golden Retrievers without a cancer diagnosis, the most important predictor was the year of study, with a higher pace observed in the earlier years and a slower pace in the later years of the study. This pattern was also observed in Golden Retrievers with cancer, where the year of study ranked as the second most important predictor.

Golden Retrievers with agility and other dog sports lifestyles were associated with faster exercise paces in both groups. Additionally, Golden Retrievers from areas with a lower percentage of Black individuals generally exhibited faster paces of exercise. Golden Retrievers without a cancer diagnosis residing in areas with larger populations of Asian, Hispanic/Latinx, and White individuals also had higher paces of exercise.

In contrast, the population demographics for Golden Retrievers with a cancer diagnosis ranked as the 6th, 9th, and 12th through 14th most important, though their specific relationship to the pace of exercise remains unclear. Water sources and climate temperature were important, as Golden Retrievers without cancer who drank primarily well water exhibited a higher pace of exercise, while Golden Retrievers with a cancer diagnosis had a lower exercise pace if given municipal water and were from hot temperature states. Figure 3A,B shows the top 15 SDH predictors of exercise pace for Golden Retrievers without cancer and those with cancer, respectively.

### 3.4. SDH for Duration of Exercise

The Poisson GPBoost model predicting the duration of exercise for Golden Retrievers without a cancer diagnosis exhibited a moderate fit, with a McFadden’s Pseudo R^2^ value of 0.15 (Table 2). In contrast, the model for Golden Retrievers with a cancer diagnosis showed poor performance, with a McFadden’s Pseudo R^2^ value of 0.06 (Table 3).

**Figure 4:**
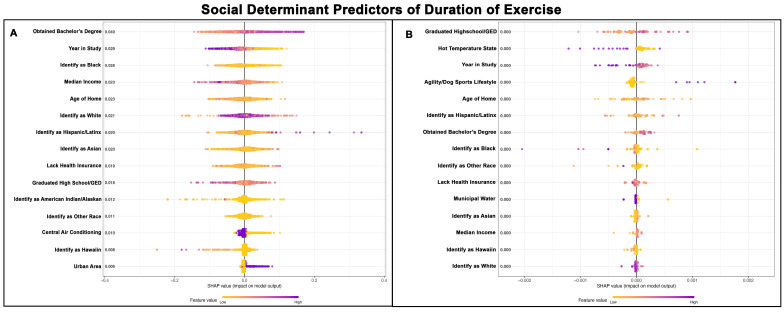
SHAP summary plots comparing SDH feature importance for duration of exercise in Golden Retrievers without a cancer diagnosis (**A**) and with a cancer diagnosis (**B**). The features are ranked top-to-bottom by mean absolute SHAP value, with more important predictors at the top and descending in order of importance. Each point represents an individual Golden Retriever, with low values represented in yellow and high values in purple.

Twelve of the top fifteen predictors of exercise duration were the same for both groups of Golden Retrievers, although their order of relative importance differed (Figure 4A,B). In both groups, Golden Retrievers from areas with a higher percentage of the population holding bachelor’s degrees exercised longer than those in areas with fewer bachelor’s degree graduates.

Notably, Golden Retrievers without cancer had longer exercise durations if they lived in areas with larger Hispanic/Latinx populations and during earlier years of the study. These patterns were not readily observable in Golden Retrievers with cancer. Other population demographic trends were not readily apparent, except that among Golden Retrievers with cancer, those from areas with higher Black populations tended to exercise for shorter durations.

In the cancer-free Golden Retriever group, dogs had shorter exercise durations if their homes lacked central air conditioning and if they lived outside of urban areas. These predictors were not in the top fifteen for Golden Retrievers with cancer. Conversely, Golden Retrievers with cancer generally had shorter exercise durations if they lived in hotter climates and were not highly involved in agility and other dog sports. These predictors were not in the top fifteen for Golden Retrievers without cancer. Further, within the cancer group, exercise duration in general increased with a higher percentage of the population holding a high school diploma or GED. While this was the top predictor, it ranked 10th in the non-cancer Golden Retriever group and the relationship between exercise duration and a high school education or GED was not readily apparent. Figure 4A,B show the top 15 SDH predictors of exercise duration for Golden Retrievers without cancer and those with cancer, respectively.

### 3.5. SDH for Frequency of Cold-Water Swimming

Both Poisson GPBoost models predicting the frequency of swimming in cold water for Golden Retrievers exhibited a poor fit with a McFadden’s Pseudo R^2^ value of 0.03 for those without a cancer diagnosis (Table 2) and McFadden’s Pseudo R^2^ value of 0.06 for those with a cancer diagnosis (Table 3).

**Figure 5:**
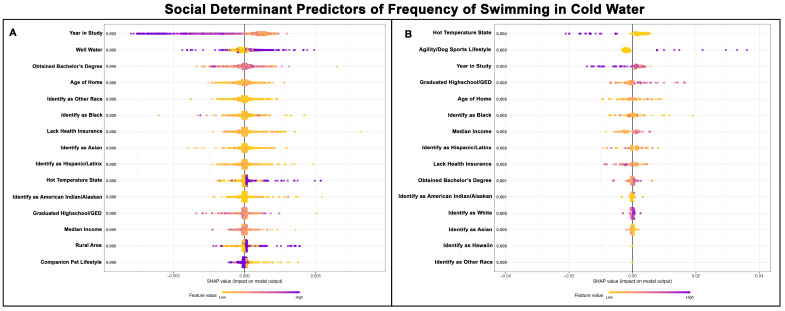
SHAP summary plots comparing SDH feature importance for frequency of cold-water swimming in Golden Retrievers without a cancer diagnosis (**A**) and with a cancer diagnosis (**B**). The features are ranked top-to-bottom by mean absolute SHAP value, with more important predictors at the top and descending in order of importance. Each point represents an individual Golden Retriever, with low values represented in yellow and high values in purple.

Golden Retrievers in both groups tended to swim in cold water more frequently during the earlier years of the study. Among Golden Retrievers without a cancer diagnosis, the second most important predictor was well water consumption, which appeared influential for both increased and decreased frequency of cold-water swimming. Opposite patterns were observed in Golden Retrievers living in hot environments—Golden Retrievers without cancer swam more frequently in cold-water swimming, while those with cancer swam less frequently in cold water. The consumption of well water, living in a rural area, and being kept as a companion pet were in the top 15 predictors for Golden Retrievers without cancer, but not for those with a cancer diagnosis. In Golden Retrievers with a cancer diagnosis, the dogs from areas with higher median incomes and greater rates of high school graduation were observed more frequently swimming in cold water, but this same pattern was not observed in Golden Retrievers without a cancer diagnosis. While demographics were important predictors for both groups, a clear pattern on the impact of cold-water swimming frequency was not readily apparent. Figure 5A,B show the top 15 SDH predictors of cold-water swimming frequency for Golden Retrievers without cancer and those with cancer, respectively.

### 3.6. SDH for Frequency of Warm-Water Swimming

**Figure 6:**
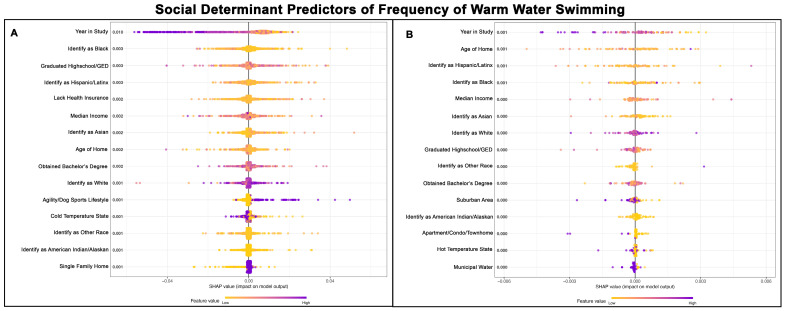
SHAP summary plots comparing SDH feature importance for frequency of warm-water swimming in Golden Retrievers without a cancer diagnosis (**A**) and with a cancer diagnosis (**B**). The features are ranked top-to-bottom by mean absolute SHAP value, with more important predictors at the top and descending in order of importance. Each point represents an individual Golden Retriever, with low values represented in yellow and high values in purple.

Both Poisson GPBoost models predicting the frequency of swimming in warm water for Golden Retrievers exhibited a poor fit, with a McFadden’s Pseudo R^2^ value of 0.06 for those without a cancer diagnosis (Table 2) and McFadden’s Pseudo R^2^ value of 0.00 for those with a cancer diagnosis (Table 3). Golden Retrievers without cancer generally who swam less frequently tended to live in a cold temperature state or during the later years of the study, while those who participated in an agility/dog sports lifestyle swam more frequently in warm water. After “year in study”, the most influential predictors were related to demographic factors and the percentage of high school graduates or GED holders in the area. Golden Retrievers without cancer living in areas with a higher percentage of uninsured individuals tended to swim more frequently in warm water. Figure 6A,B show the top 15 SDH predictors of warm-water swimming frequency for Golden Retrievers without cancer and those with cancer, respectively.

### 3.7. SDH for Activity Level

**Figure 7:**
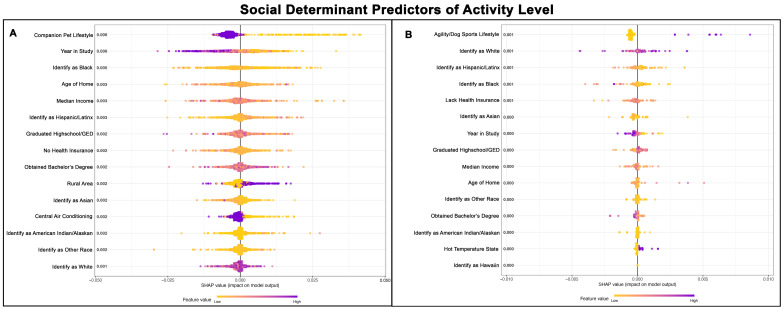
SHAP summary plots comparing SDH feature importance for activity level of Golden Retrievers without a cancer diagnosis (**A**) and with a cancer diagnosis (**B**). The features are ranked top-to-bottom by mean absolute SHAP value, with more important predictors at the top and descending in order of importance. Each point represents an individual Golden Retriever, with low values represented in yellow and high values in purple.

The Poisson GPBoost model predicting the activity level of Golden Retrievers with and without a cancer diagnosis exhibited a poor fit, with a McFadden’s Pseudo R^2^ value of 0.01 (Table 2 and Table 3). Figure 7A,B show the top 15 SDH predictors of activity level for Golden Retrievers without cancer and those with cancer, respectively.

### 3.8. SDH for Duration of Sun Exposure

**Figure 8:**
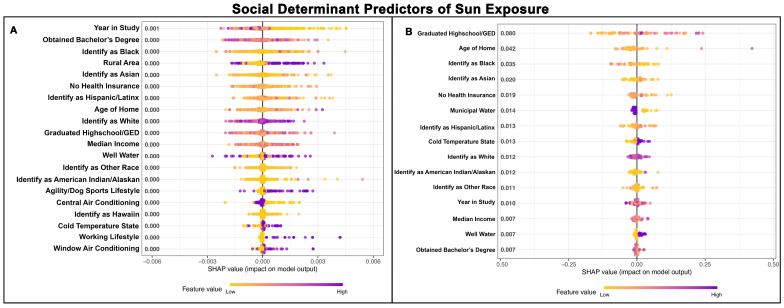
SHAP summary plots comparing SDH feature importance for the duration of sun exposure of Golden Retrievers without a cancer diagnosis (**A**) and with a cancer diagnosis (**B**). The features are ranked top-to-bottom by mean absolute SHAP value, with more important predictors at the top and descending in order of importance. Each point represents an individual Golden Retriever, with low values represented in yellow and high values in purple.

The Poisson GPBoost model predicting the length of sun exposure of Golden Retrievers with and without a cancer diagnosis exhibited a poor fit, with a McFadden’s Pseudo R^2^ value of 0.00 (Table 2 and Table 3). Figure 8A,B shows the top 15 SDH predictors of sun exposure length for Golden Retrievers without cancer and those with cancer, respectively.

## 4. Discussion

Over the initial eleven years of the GRLS, only a single peer-reviewed study incorporated SDHs [37]. This helps to highlight the larger research gap that exists surrounding SDHs and health outcomes in companion animals—it mostly remains unknown. We selected SDHs recorded by the owners along with zip code-level demographics, income, and education from the US Census Bureau ACS. The selected SDHs can be grouped by five commonly recognized domains of social determinants of health: (1) economic stability, (2) access to and quality of health care, (3) access to and quality of education, (4) neighborhood and built environment, and (5) social and community context [38].

In evaluating economic stability as an important domain of health outcomes, our study incorporated several key socioeconomic predictors. These included median household income (source: ACS), type of home (e.g., single family unit, apartment, etc. source: MAF Annual Owner Survey), and housing amenities (source: MAF Annual Owner Survey), with particular attention to central air-conditioning as a proxy for home quality and socioeconomic status.

Evidence from prior research in human medicine links lower socioeconomic status with poorer outcomes in cases with lymphoma [39]. Smith et al., 2012 [40] reported that individuals from lower socioeconomic backgrounds were significantly more likely to be diagnosed at a later stage of Hodgkin lymphoma, a factor that can negatively affect prognosis and treatment outcomes. In parallel, individuals with lower socioeconomic status, especially women, are more likely to engage in lower levels of physical activity compared to individuals with higher socioeconomic status [41]. Physical activity itself is a well-established protective factor, with higher activity levels associated with a reduced risk of lymphoma development [42].

Together, this literature informed our analysis approach. We proactively constructed separate models for the cancer-free and cancer-diagnosed Golden Retriever populations for each physical activity variable. By keeping the two populations separate, it allowed us to evaluate whether associations between socioeconomic determinants and canine physical activity patterns differed between cancer-free dogs and dogs diagnosed with cancer.

Recognizing that the risk of canine lymphoma is multifactorial, influenced by both genetic predispositions and environmental exposures, and often associated with pet owner behaviors, it is not surprising that socioeconomic factors can also impact the diagnosis and management of cancer disease in dogs [43]. Confirmed cases of multicentric lymphoma were more commonly observed among pet owners residing in more affluent areas [44]. Additionally, Stoewen et al. examined the factors influencing veterinarians’ decision to refer patients to a veterinary oncologist, and while several variables play a key role, including distance to the specialty referral center, confidence in the referral practice, and experience with chemotherapy treatments, two key factors highlighted were the owner’s financial capacity and the strength of the human–animal bond [45]. These findings are consistent with the results of our study, particularly in cases involving a cancer diagnosis, and provide further support for our hypothesis that socioeconomic factors, such as higher income, may influence owner behaviors that shape a dog’s daily activity patterns.

Another study supporting our hypothesis and findings found neighborhood disadvantage, defined as a combination of ACS variables related to income, poverty line, employment, education, and household composition, was found to significantly negatively impact on-leash dog walking [46]. While most studies investigating dog walking and physical activity focus on comparing dog owners to nondog walkers, a few studies suggest that dog walking is determined by social support [47,48], time availability [49], and neighborhood [15,46,50]. Our best performing models, year in study and frequency of exercise, both revealed that neighborhood-linked variables such as age of home, access to health insurance, and median household income impacted both canine physical activity patterns and continued enrollment in the study. Age of home was consistently highly associated with both of these models, suggesting that neighborhood context and residential stability translate into measurable variation in activity levels among dogs, regardless of cancer status, and impact the participation in longitudinal studies.

While background education and information giving play a critical role in understanding a cancer diagnosis, they can also significantly influence an owner’s willingness to pursue treatment and adhere to veterinary recommendations [45]. In veterinary medicine, adherence is closely tied to both the perceived role of the animal within the household and the owner’s understanding of the diagnosis and its implications for the animal’s quality of life [51]. In our study, Golden Retrievers without a cancer diagnosis whose primary role was that of a companion pet were more likely to remain enrolled for longer periods and exhibited longer lifespans, suggesting that owner engagement and study adherence may be linked to perceptions of the human–animal bond.

Across models for both dogs with and without cancer diagnoses, owners with higher levels of education (source: ACS), particularly those holding a bachelor’s degree, were more likely to remain engaged in the study and to exercise more frequently. This suggests that educational background may be an important predictor of continued participation and adherence in veterinary medicine and supports our hypothesis that owners with higher education levels would self-report higher levels of canine physical activity and improved health outcomes.

Access to veterinary care is financially challenging, with a recent study finding 52% of pet parents not seeking veterinary care or declining veterinary care, in large part due to the inability to afford the cost [52]. Socioeconomic factors such as income and education also appear to influence broader patterns of health-related decision-making. For example, owners who obtained health insurance for their pets had at least a high school education and an income of $55,000 or more, and those owners with pet health insurance spent more on their pet’s healthcare and unexpected expenses such as cancer treatment compared to non-insured owners [53], highlighting that both finances and education were important. While it is unknown if canine cancer diagnosis and treatment success are linked to health insurance, studies consistently document lower survival in humans afflicted with lymphoma, osteosarcomas, and other cancers in populations who lack health insurance or are insured through Medicare [54,55,56]. In our study, the rate of health insurance coverage (source: ACS) was consistently predictive for canine physical activity patterns across many of the models. Most pronounced was the finding that owners living in areas with a high percentage of individuals who lacked health insurance were found to more frequently exercise their Golden Retrievers. While the relationship between health insurance coverage and physical activity has not been well characterized in veterinary medicine nor human medicine, the few human studies suggest the interaction between health insurance coverage and exercise is complex and sometimes counterintuitive. A single study by Foute et al. (2015) found that patients with health insurance with a drug plan were less likely to exercise regularly [57]. While those whose health care providers discuss physical activity and exercise typically increase their activity levels, most health care providers infrequently discuss physical activity and exercise with their patients [58] and, combined with other barriers such as culture and environment, there may be a complex interaction of factors that should be explored by future studies.

Previous studies have also shown that dogs living in industrial areas or in households where potentially hazardous chemicals are used are at an increased risk for cancer, particularly canine lymphoma [59]. In our study, we identified several interesting environmental factors (source: MAF Annual Owners Survey). For example, swimming frequency in both cold and warm water emerged as a relevant physical activity outcome, with Golden Retrievers without a cancer diagnosis exhibiting more frequent swimming in cold water compared to dogs diagnosed with cancer. Among dogs without a cancer diagnosis, residing in rural areas and drinking well water were associated with higher reported swimming frequency. These findings reflect SDH impacting physical activity patterns differently based upon the canine population’s cancer status. However, it should be noted it is well established that exposure to contaminated tap water as well as environmental pollutants can increase the risk of lymphoma and other cancers in dogs [39].

Beyond the financial challenges, it is well established that different populations face both economic and cultural barriers to healthcare [60]. Consistently, the percentage of the population that identified as either Asian, Black, Hispanic/Latinx, or White (source: ACS) were identified as important to the model. Our results were unclear as to how the ethnicities were linked to the dependent variable, and this suggests that there may be complex interactions between ethnicities and the other social determinants for models with an overall good fit; however, some of our models demonstrated an overall poor fit—with little of the observed variability explained. While this is a limitation of our study, it also further supports that there may be complex interactions between ethnicities and variables not investigated. A limitation of our study was not assessing complex interactions. Future studies should take this into consideration, and researchers should consider developing a validated, composite score system that accounts for multiple indicators of a sole SDH when designing studies focused on assessing the impact of SDHs on longitudinal data. It is possible this could help improve model performance. The data source should also be considered, as zip code-level SDHs may be less accurate than owner-reported SDHs. Additionally, some variables, such as “identify as White” and “companion pet lifestyle”, were overrepresented in the data and indicate a potential bias when recruiting participants or a hesitancy in participating in the study. This bias is often seen in the recruitment of human participants for health research studies [61], and innovative ways to increase participation of underrepresented populations should be considered in future companion animal studies.

In summary, our study results highlight SDHs and explain several key factors, including year in study, frequency, duration, and pace, as important predictors for health outcomes in Golden Retrievers, both with and without a cancer diagnosis. However, further research is needed to clearly establish the dose and response of their influence while also accounting for the interrelationships among these factors.

## Figures and Tables

**Table 1:** Summary of SDH variables incorporated into the study and the source of the data. Variables were selected based upon availability from the GRLS study and available zip code-level data that has been shown to impact human physical activity and/or obesity rates.

Data Source	Social Determinant of Health
Morris Animal Foundation	lifestyle category, heating systems, cooling systems, home type, home age, primary state of residence, primary zip code of residence, geographical delineation (e.g., rural environment), water source home type
National Oceanic and Atmospheric Administration (NOAA)	State average temperature
US Census Bureau American Community Survey	Median income, mean income, bachelor’s degree holders, high school graduates/GED graduates, individuals without home insurance, zip code demographics

**Table 2:** Poisson model performance metrics for dogs without cancer.

Target Variable	MSE	RMSE	McFadden’s Pseudo R^2^
Year in study	1.70	1.30	0.70
Frequency	1.50	1.22	0.94
Pace	1.51	1.23	0.24
Duration	1.50	1.23	0.15
Frequency of cold-water swimming	1.12	1.06	0.03
Frequency of warm-water swimming	1.22	1.05	0.06
Activity level	0.24	0.49	0.01
Sun exposure duration	0.21	0.46	0.00

**Table 3:** Poisson model performance metrics for dogs diagnosed with cancer.

Target Variable	MSE	RMSE	McFadden’s Pseudo R^2^
Year in study	1.63	1.28	0.80
Frequency	1.51	1.23	0.94
Pace	1.50	1.23	0.07
Duration	1.52	1.23	0.06
Frequency of cold-water swimming	1.57	1.25	0.06
Frequency of warm-water swimming	1.35	1.16	0.00
Activity level	0.23	0.48	0.01
Sun exposure duration	0.14	0.38	0.00

## Data Availability

Restrictions apply to the availability of these data. All physical activity data used in this study is available from Morris Animal Foundation Data Commons and all SDH data is available by request from Morris Animal Foundation Golden Retriever Lifetime Study Data Team. Data are available at https://datacommons.morrisanimalfoundation.org (accessed on 24 May 2023) of Morris Animal Foundation. The R code for the analysis is available in the Github Repository MicroBatVet/GRLS (https://github.com/MicroBatVet/GRLS, accessed on 27 January 2026).

## Abbreviations

The following abbreviations are used in this manuscript:

MAF	Morris Animal Foundation
GRLS	Golden Retriever Lifetime Study
MSE	Mean squared error
RMSE	Root mean squared error
SDH	Social determinant of health

## Appendix A

Final model parameters for each GPBoost model are in Table A1, Table A2, Table A3, Table A4, Table A5, Table A6, Table A7 and Table A8.

**Table A1:** The best parameters for the SDH GPBoost models when assessing the year of study.

Parameter	No Cancer Diagnosis	Cancer Diagnosis
Learning rate	0.1	0.01
Min_data_in_leaf	100	1
Max_depth	−1	35
Num_leaves	512	512
Lambda_l2	0	1
Max_bin	250	250
Lin_search_step_length	TRUE	TRUE
Tree_learner	serial	serial
Number of iterations	100	100

**Table A2:** The best parameters for the SDH GPBoost models when assessing the frequency of exercise.

Parameter	No Cancer Diagnosis	Cancer Diagnosis
Learning rate	0.1	0.001
Min_data_in_leaf	100	1
Max_depth	−1	15
Num_leaves	512	64
Lambda_l2	0	1
Max_bin	250	500
Lin_search_step_length	TRUE	TRUE
Tree_learner	serial	serial
Number of iterations	10	21

**Table A3:** The best parameters for the SDH GPBoost models when assessing the pace of exercise.

Parameter	No Cancer Diagnosis	Cancer Diagnosis
Learning rate	0.1	0.01
Min_data_in_leaf	100	1
Max_depth	−1	35
Num_leaves	512	512
Lambda_l2	0	1
Max_bin	250	250
Lin_search_step_length	TRUE	TRUE
Tree_learner	serial	serial
Number of iterations	10	10

**Table A4:** The best parameters for the SDH GPBoost models when assessing the duration of exercise.

Parameter	No Cancer Diagnosis	Cancer Diagnosis
Learning rate	0.1	0.001
Min_data_in_leaf	100	1
Max_depth	−1	45
Num_leaves	512	32
Lambda_l2	0	1
Max_bin	250	250
Lin_search_step_length	TRUE	TRUE
Tree_learner	serial	data_parallel
Number of iterations	10	5

**Table A5:** The best parameters for the SDH GPBoost models when assessing the frequency of cold-water swimming.

Parameter	No Cancer Diagnosis	Cancer Diagnosis
Learning rate	0.1	0.001
Min_data_in_leaf	100	1
Max_depth	−1	45
Num_leaves	512	32
Lambda_l2	0	1
Max_bin	250	250
Lin_search_step_length	TRUE	TRUE
Tree_learner	serial	data_parallel
Number of iterations	10	5

**Table A6:** The best parameters for the SDH GPBoost models when assessing the frequency of warm-water swimming.

Parameter	No Cancer Diagnosis	Cancer Diagnosis
Learning rate	0.0001	0.01
Min_data_in_leaf	1	1
Max_depth	35	−1
Num_leaves	512	128
Lambda_l2	0	0
Max_bin	500	250
Lin_search_step_length	TRUE	TRUE
Tree_learner	serial	serial
Number of iterations	1000	10

**Table A7:** The best parameters for the SDH GPBoost models when assessing the activity level.

**Parameter**	**No Cancer Diagnosis**	**Cancer Diagnosis**
Learning rate	0.0001	0.0001
Min_data_in_leaf	10	10
Max_depth	−1	−1
Num_leaves	1024	1024
Lambda_l2	1	1
Max_bin	150	150
Lin_search_step_length	FALSE	FALSE
Tree_learner	serial	serial
Number of iterations	100	100

**Table A8:** The best parameters for the SDH GPBoost models when assessing the sun exposure duration.

**Parameter**	**No Cancer Diagnosis**	**Cancer Diagnosis**
Learning rate	0.0001	0.015
Min_data_in_leaf	10	10
Max_depth	−1	15
Num_leaves	1024	512
Lambda_l2	1	0
Max_bin	150	50
Lin_search_step_length	FALSE	FALSE
Tree_learner	serial	serial
Number of iterations	100	100
